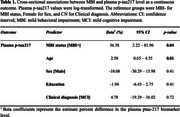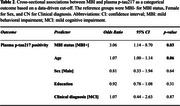# Plasma *p*‐tau217 in relation to mild behavioral impairment: implications for early detection of Alzheimer's disease

**DOI:** 10.1002/alz70856_103819

**Published:** 2025-12-25

**Authors:** Maryam Ghahremani, Rebeca Leon, Eric E. Smith, Zahinoor Ismail

**Affiliations:** ^1^ Hotchkiss Brain Institute, University of Calgary, Calgary, AB, Canada; ^2^ Department of Clinical Neurosciences, University of Calgary, Calgary, AB, Canada; ^3^ University of Calgary, Calgary, AB, Canada

## Abstract

**Background:**

The 2024 NIA‐AA revised criteria define Alzheimer's disease (AD) biologically through core biomarkers amyloid‐beta (Aβ) and tau. While cognitive function remains central to the clinical evaluation of early‐stage dementia, mild neurobehavioral changes may coexist and even precede cognitive decline. Mild behavioral impairment (MBI), marked by late‐onset persistent neuropsychiatric symptoms (NPS), has emerged as a potential early indicator of dementia risk. While MBI is associated with well‐established Aβ and tau biomarkers, the association with plasma *p*‐tau217, a novel blood‐based biomarker with high accuracy for AD‐related pathology, remains unexplored. Here, we investigated the association between MBI and plasma *p*‐tau217 levels in older adults with normal cognition or mild cognitive impairment (MCI) from the Alzheimer's Disease Neuroimaging Initiative.

**Method:**

NPS scores were obtained from the Neuropsychiatric Inventory, with MBI status (MBI+/‐) determined over two consecutive visits to operationalize the MBI symptom persistence criterion. Participants without plasma ptau‐217 data prior to dementia diagnosis were excluded. Linear regression modeled the association between NPS status and *p*‐tau217 level as a continuous variable outcome. Additionally, logistic regression modeled the association between NPS status and *p*‐tau217 positivity status, using a study‐specific cut‐off derived using Gaussian mixture modeling. Models adjusted for age, sex, education, and cognitive diagnosis.

**Result:**

Plasma *p*‐tau217 levels and NPS status were available in 101 participants (50.5% MCI; mean age 72.0±6.5; 44.6% female). Participants with MBI had significantly higher plasma *p*‐tau217 levels (Beta=36.4%; 95%CI: 2.2‐82.0, *p* = 0.04) (Table 1) and higher odds of being *p*‐tau217 positive (OR=3.06, 95%CI: 1.14‐8.70, *p* = 0.03), relative to MBI‐ participants (Table 2).

**Conclusion:**

Findings add to the evidence base that appropriately measured behavioural symptoms can represent AD proteinopathies, supporting the role of MBI in AD risk stratification. The link between MBI and elevated plasma *p*‐tau217 levels highlights the potential utility of MBI for early AD detection and more efficient clinical trial design by utilizing MBI assessment at screening to identify high‐risk individuals for biomarker positivity.